# Regulatory link mapping between organisms

**DOI:** 10.1186/1752-0509-5-S1-S4

**Published:** 2011-05-04

**Authors:** Rachita Sharma, Patricia A Evans, Virendrakumar C Bhavsar

**Affiliations:** 1 Faculty of Computer Science, University of New Brunswick, Fredericton, NB E3B5A3, Canada

## Abstract

**Background:**

Identification of gene regulatory networks is useful in understanding gene regulation in any organism. Some regulatory network information has already been determined experimentally for model organisms, but much less has been identified for non-model organisms, and the limited amount of gene expression data available for non-model organisms makes inference of regulatory networks difficult.

**Results:**

This paper proposes a method to determine the regulatory links that can be mapped from a model to a non-model organism. Mapping a regulatory network involves mapping the transcription factors and target genes from one genome to another. In the proposed method, Basic Local Alignment Search Tool (BLAST) and InterProScan are used to map the transcription factors, whereas BLAST along with transcription factor binding site motifs and the GALF-P tool are used to map the target genes. Experiments are performed to map the regulatory network data of *S. cerevisiae* to *A. thaliana* and analyze the results. Since limited information is available about gene regulatory network links, gene expression data is used to analyze results. A set of rules are defined on the gene expression experiments to identify the predicted regulatory links that are well supported.

**Conclusions:**

Combining transcription factors mapped using BLAST and subfamily classification, together with target genes mapped using BLAST and binding site motifs, produced the best regulatory link predictions. More than two-thirds of these predicted regulatory links that were analyzed using gene expression data have been verified as correctly mapped regulatory links in the target genome.

## Background

A transcriptional gene regulatory network [1] represents a collection of regulatory elements, which are target genes and transcription factors, interacting with each other in a cell to regulate the rate of transcription of genes in the network. A regulatory relationship in a gene regulatory network consists of a transcription factor, a target gene, and the type of regulatory relationship between the regulatory elements, either positive or negative.

These regulatory relationships in a network can help answer current biological questions, such as the identification of genes and proteins related to various diseases, and are useful in novel drug design and development [2]. These regulatory relationships can also be useful in understanding the differences in gene regulation between different organisms. Since it is critical to study how genes are involved in regulation, or the way they are themselves regulated by other genes, the determination of gene regulatory networks is extremely important for understanding gene regulation by identifying these genes and their relationships.

Significant time and resources are required for the experimental determination of these gene regulatory networks. Experimental techniques such as gene-knockout experiments [3] are extremely time-consuming and in many cases inadequate to identify a regulatory network for an organism at the genome level. The amount of genetic information available for newly sequenced genomes is increasing exponentially, and therefore it is essential to develop methods to bridge the gap and infer regulatory networks for these new genomes. Several computational models have been used to represent gene regulatory pathways for model organisms using gene expression data, and some models incorporate additional available biological knowledge. These models include Boolean Networks [4,5], Bayesian Networks [6-8], Differential equation models [9] and Hybrid Petri Nets [10]. Recently, many techniques have started incorporating additional data, such as protein-protein interaction data, protein-DNA interaction data, and binding site data, along with the gene expression data to get more accurate gene regulatory networks. Additionally, many methods are now focusing on using time-course behavior of gene expression along with available biological knowledge. Some of the above techniques have been discussed in a comparative review [11,12] of determination of gene regulatory networks. The above mentioned techniques cannot be used for most non-model organisms due to data sparseness.

Some methods, as discussed in [11], are building on gene expression techniques by integrating a variety of different information sources in order to improve their results. Recent work [13] has pushed the uses of these methods further, using expression-based network inference for multiple related organisms and pattern discovery techniques to find transcription binding site motifs. This type of extension of regulatory network prediction is very useful to explore organisms for which a significant amount of data is available. However, to use an organism as a model, once this exploration is done, we need to be able to determine how much of its information can be transferred to related organisms about which we may know very little. These aforementioned methods, which infer regulatory networks either entirely or primarily from gene expression data, work well with the large amounts of gene expression data that are readily available for model organisms, or for other popular organisms. Model organisms are investigated thoroughly by biologists, by virtue of being simpler and easy to manipulate and having short life cycles. Some other organisms are more complex, but are similar to model organisms in their popularity, leading to significant quantities of data being available for them as well. The information from these model organisms can be mapped to newly sequenced organisms about which less is known, referred to as non-model organisms. These non-model organisms in many cases have very limited data available, which prevents the success of the previously discussed methods for inferring regulatory relationships and other information.

There have been two methods developed, P-MAP [14] and another one used in KEGG [15], that use a regulatory network of a model organism to infer a regulatory network for a non-model organism. However, these methods are limited in their usability for mapping regulatory pathways. The mapping in P-MAP, taken as a constrained minimum spanning tree problem, is based on keeping overall sequence similarity between mapped gene pairs and preserving the co-regulated gene structures in the predicted pathway. The P-MAP algorithm is also limited in that it does not predict the direction and type of regulatory relationships between the genes. In KEGG [15], organism specific pathways are constructed based on ortholog identifier assignments in the GENES database. It relies on previously determined information about homologous genes, and it does not consider any other data.

This paper determines the amount of gene regulatory information that can be mapped from a model organism (source genome) to a non-model organism (target genome), based only on gene and promotor sequences and transcription factor binding site motifs. The use of gene expression data for testing also suggests how it could be incorporated into the technique to improve results while still not requiring large quantities of experimental data. This technique is thus useable for a variety of different organisms for which there is insufficient data to infer regulation using gene expression data. Due to the extreme differences between the minimal amount of data used in this method and the large quantities of data, particularly expression data, that are used by other techniques, it is not appropriate to directly compare the results. Identification of regulatory information for a non-model organism will help the biologists investigate any new organism at the genome level and provide information about the common regulatory relationships between the two genomes. The method in this paper involves mapping the transcription factors (TFs), target genes (TGs) and their regulatory relationships from one genome to another. Gene expression data available for the target genome is used in this paper to evaluate the results due to insufficient reliable regulatory network information being available for testing. A set of rules is established to use the information from the gene expression experiments to analyze the regulatory relationships predicted for the target genome.

In the proposed method, any model organism with an available experimentally confirmed regulatory network and transcription factor binding site (TFBS) motif information can be used as the source genome. However, a model organism closer to the target genome is preferable, since evolutionarily closer organisms will tend to have more similar regulatory relationships. The target genome can be any non-model organism with available nucleotide sequence data. For experimentation and testing purposes *S. cerevisiae* and *A. thaliana* have been used in this paper as source and target genomes, respectively. *S. cerevisiae* is a model organism with all the required information required for a source genome. *A. thaliana*, not being a non-model organism, is used as the target genome for experimentation purposes only, so that the gene expression data available for *A. thaliana* can be used for analysis and verification of the mapped regulatory relationships determined using the proposed method. *A. thaliana* is also a suitable test organism to investigate regulatory inference for plants, for which there are far fewer well-investigated examples and thus are likely targets of the technique presented in this paper. While closer organisms are preferred, *S. cerevisiae* and *A. thaliana* are used as the source and target genomes since there is no suitable pair of organisms that are evolutionarily close that have both sufficient regulatory network and gene expression information available for verification. This non-ideal situation can nevertheless be common for many non-model organisms, due to the limited availability of well-investigated model organisms, and the results in this paper thus identify the proportion of regulatory relationships that can be mapped from an evolutionarily distant model organism.

The mapping of TFs between genomes based on evolutionary distance has been investigated in our previous work using bacterial genomes [16]. We considered 14 bacterial genomes, and for each of them, based on the ROC curve analysis, we determined the best e-value threshold for BLAST [17] and the best model organism to use, between *Bacillus subtilis* and *E. coli*, to map regulatory information. The results were evaluated based on sensitivity, PPV and Receiver Operating Characteristic (ROC) curve to determine the best combination to use for every target bacterial genome used. The PPV ratio (accuracy) and sensitivity is high for suitable e-value thresholds. The sensitivity decreases for lower e-value thresholds, whereas the accuracy decreases with increase in e-value thresholds. Therefore, the best e-value thresholds provide a good balance between losing the true TFs and predicting more non-factors. The best e-value thresholds determined for all examined bacterial genomes and both source organisms were either the same or very close to each other. Therefore, it is likely that the best e-value determined may also work well for TF mapping between other set of genomes. A key finding was that using the correct e-value threshold was just as, and in some cases more, important than using the closer model organism; this result provides a foundation for this exploration of methods to map regulatory links using more distantly related organisms.

## Methods

### Transcription factor mapping

Mapping TFs from one genome to another involves finding similar protein sequences in the target genome performing similar functions. The sequence alignment tool BLAST [17] and the functional similarity tool InterProScan [18] are used for TF mapping, which is the first step in regulatory link mapping.

Similar regions in the gene sequences tend to indicate similar structure or function preserved by evolution. Additionally, specific conserved motifs in the protein sequences, called protein signatures, define the structure and function of the proteins. Therefore, similar protein sequences with common protein signatures generally perform similar functions and belong to the same functional group. InterPro [19] is a non-redundant database that integrates the commonly used protein signature databases. In this paper, the PANTHER (Protein ANalysis THrough Evolutionary Relationships) database [20] and its corresponding scanning tools, BLAST and hmmsearch, are used within InterProScan. The PANTHER database consists of protein sequences classified into families and subfamilies with similar function based on published experimental evidence and evolutionary relationships.

**Figure 1:**
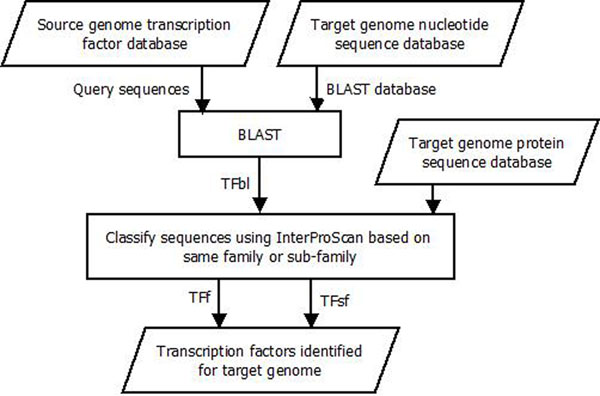
**Method to map transcription factors from a source to a target genome** Three sets of transcription factors are determined for the target genome: TFbl, TFf and TFsf. These result sets are obtained based on sequence similarity, protein family classification and protein subfamily classification.

Figure 1 shows that first the BLAST tool is executed, using the target genome nucleotide sequence data as the BLAST database and the source genome TFs as the query sequences. The BLAST results are those target genome sequences that are similar to the source genome TFs. Three different variations can then be followed in this method: the BLAST results can be used without further refinement, or they can be refined further by InterProScan using either family or subfamily classification. Three result sets of predicted TFs are determined based on sequence similarity (TFbl), same protein family (TFf) and subfamily classification (TFsf). Not all the TFs can be mapped from one genome to another genome, even when these genomes are evolutionarily very close. Moreover, there is no knowledge of the number of TFs that should be mapped from one genome to another. Hence, the number of confirmed TF mappings is based on the definition of correct mapping used. Each of the results from the method are analyzed to find out if a sequence predicted as a TF is an experimentally verified TF of the target genome or not.

The result sets are compared and analyzed using a binary classifier [21]. The results are classified into four groups, true positives (TP), false positives (FP), true negatives (TN) and false negatives (FN). The TPs are the predicted TFs that are present in the available TF database of the target genome. FPs are the predicted TFs that are not present in the available TF database. The TFs that are present in the available TF database of the target genome but have not been identified by the method are the FNs. The sequences that are not present in the available TF database and are discarded by the method are TNs.

### Target gene mapping

Mapping TGs from one genome to another, for a particular TF in a regulatory network, involves finding similar TGs in the target genome. These similar TGs should have the same function as the source genome TGs. Similar function is important in order to have the same type of regulatory relationship in the target genome as the regulatory relationship being mapped from in the source genome. Since TGs may or may not produce proteins, they cannot be grouped based on similar protein signatures, but the BLAST tool can be used to find highly similar nucleotide sequences that will tend to have similar function.

Additionally, being part of the gene regulation process, the TF of a regulatory link binds to the binding site containing the TF binding site (TFBS) motif, which is generally located upstream of the TG sequence [22]. The TFs look for specific motifs (patterns) in the binding site regions of the TGs based on the type and family of the TF. Different TFs have different sets of TFBS motifs that they look for in order to regulate their TGs. Hence, the TG being regulated by a certain TF in the source genome will tend to have one of the specific TFBS motifs of that TF in its binding site region. The binding sites of the mapped TGs in the target genome will also tend to contain one of the TFBS motifs of the source genome TF from the regulatory link being mapped. If the TFBS motif information is not available for the source genome, then the TFBS motifs can be located using one of the available methods used for finding TFBS motifs. The current genetic algorithms for identifying TFBS motifs use position-led and consensus-led representations independently whereas GALF-P (Genetic Algorithm with Local Filtering and adaptive post-processing techniques) [23] combines both representations and uses local filtering to decrease false positives and improve efficiency.

**Figure 2:**
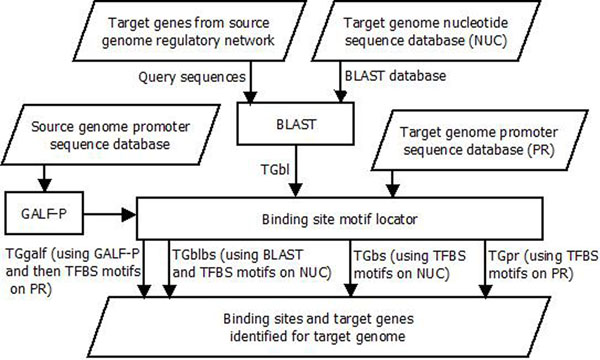
**Method to map target genes from a source to a target genome** Five sets of target genes are identified for the target genome: TGbl, TGbs, TGblbs, TGpr and TGgalf. These sets are determined based on sequence similarity, searching TFBS motifs and sequence similarity combined with locating TFBS motifs.

Figure 2 shows the second step of regulatory link mapping where the TGs are mapped from one genome to another. For a particular TG in a regulatory link, the BLAST tool is used to find the similar TGs in the target genome. Target genome nucleotide sequence data is the BLAST database, and the source genome TGs from the regulatory network being mapped are the query sequences. The output of BLAST is a set of similar TGs that are then the input for the next step of locating the binding sites. The binding site motif locator searches for the TFBS motifs of the source genome TF in the similar TG sequences found in the target genome. Finally, the genes that are similar (found using BLAST) and have the right TFBS motifs (found using the Binding site motif locator) are determined to be the predicted TGs for the target genome. This set of predicted TGs are termed TGblbs. TGs are also identified using only the BLAST tool, the first step in this TG mapping method, producing set TGbl. Another set of predicted TGs named TGbs is determined by only searching the TFBS motifs, the second step in this method. The promoter sequence data of the target genome is searched with the TFBS motifs to obtain the predicted TGs in set TGpr. The final set TGgalf of predicted TGs is determined by using the GALF-P tool to identify the TFBS motifs, followed by searching for these motifs in the target genome promoter sequence database.

The result sets are evaluated by comparing the mapped TGs with the available binding site data for *A. thaliana* to determine the number of mapped TGs predicted as the correct TGs in the target genome. A binary classifier is used to analyze these results in the same way as the mapped TFs are analyzed in the previous subsection.

### Mapped regulatory elements integration

The final step of mapping a regulatory network from the source genome is to integrate the mapped regulatory elements to obtain the regulatory links of the target genome. It is crucial to find out if the predicted TG in the target genome is correctly linked to the right TF by the link mapped from the source genome.

**Figure 3:**
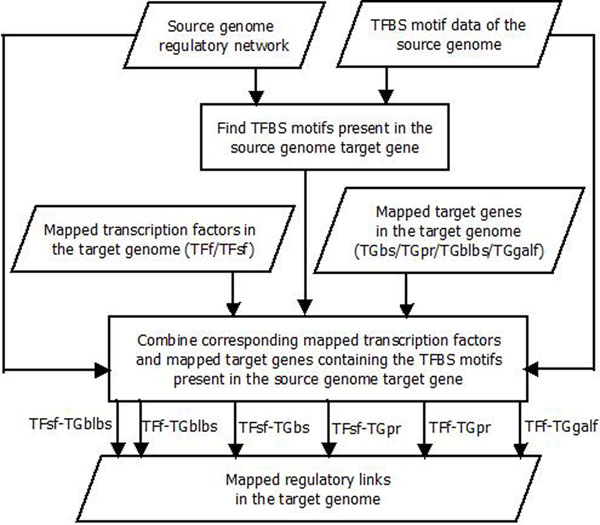
**Method to integrate mapped regulatory elements for the target genome** Two sets of transcription factors (TFf and TFsf) are integrated with four sets of target genes (TGblbs, TGbs, TGpr and TGgalf) to determine six sets of regulatory links for the target genome: TFsf- TGblbs, TFf- TGblbs, TFsf- TGbs, TFsf-TGpr, TFf-TGpr and TFf-TGgalf.

Two regulatory links from two different genomes tend to be similar if the two TFs from these links bind to the same motifs in the TGs, implying that these TFs might regulate the TGs in a similar way. Hence, the TFBS motif of the TF present in the TG of the source genome should also be present in the TG of the target genome. In this final step of regulatory network mapping shown in Figure 3, for every link in the source genome regulatory network, the corresponding predicted TFs are located among the mapped TFs identified in the first step of network mapping. The TFBS motifs of the source genome TF are searched in its TG. The TFBS motifs that are present in the source genome TG are then searched in the corresponding mapped TG nucleotide sequences of the target genome. Finally, these TFs and the TGs, which contain the specific TFBS motifs, are combined to obtain the target genome regulatory links.

Six sets of predicted regulatory links are identified using this method. The first set TFsf-TGblbs is obtained by integrating the predicted TF set TFsf, combining BLAST similiarity and subfamily classification, and the predicted TG set TGblbs, combining BLAST similarity and TF binding sites. The set TFf found using BLAST similarity and family classification is integrated with the set TGblbs to identify the second predicted regulatory links set TFf-TGblbs. The third set TFsf-TGbs is identified by combining the mapped TFs from the set TFsf and the mapped TGs from the set TGbs, found using binding sites alone. The fourth and fifth sets, TFsf-TGpr and TFf-TGpr, are identified by combining the target genes in TGpr from searching promotor regions for binding site motifs with the transcription factors found with protein subfamily and family classification, respectively. The sixth and final set TFf-TGgalf is identified by combining the transcription factors using family classification with the target genes TGgalf found through locating motifs determined by GALF-P in the promotor sequences.

### Regulatory link confirmation

The mapped regulatory links of the target genome cannot be confirmed easily because very limited gene regulatory information is available for the model organisms that can be used to verify the predicted regulatory links. Gene expression data can be used for analyzing the mapped regulatory links of the target genome and is available for verification.

Gene expression data contains expression levels of some of the genes investigated for a genome in any gene expression experiment. Different experiments for the genome contain different expression levels of the genes depending on the experimental conditions. On the basis of the expression value, a gene is considered expressed or not in any given experiment. The expression levels of the TF and the TG from a predicted regulatory link in different experiments can be investigated to determine if the expression data supports the regulatory relationship in the predicted link. In the first step, the predicted regulatory links' data and the gene expression data from a few experiments for the target genome is used to check if the expression values of the regulatory elements are present in the experiments. For any predicted regulatory link, the information of the regulatory elements, as expressed (Yes), not expressed (No) or absent (ab), in every gene expression experiment is collected. A set of rules is established to verify the regulatory links based on regulation type, TF expression and TG expression in the gene expression experiments as shown in Table 1. Using these rules, the gene expression experiments for every predicted regulatory link are distributed into three groups, Confirming (*C*), Contradictory () and Neutral (*N*). The number of elements in the Confirming group (*c*) for a predicted regulatory link represents the number of gene expression experiments that verify that the predicted regulatory link is correctly mapped in the target genome. The number of gene expression experiments that contradict the regulatory relationship in the predicted regulatory link act as the number of elements in the Contradictory group () for that regulatory link. The number of elements in the Neutral group (*n*) for a predicted regulatory link corresponds to the number of gene expression experiments that neither confirm nor contradict that regulatory link but can provide additional information. The gene expression experiments for a predicted regulatory link are ignored if they have the expression values of the TFs and TGs present in the experiments but neither support nor contradict that regulatory link and also do not provide any further information. If the expression information of a TF or TG from a regulatory link is absent in a gene expression experiment, then that experiment is ignored for that regulatory link, since nothing can be inferred unless the expression information of all regulatory elements is available.

**Table 1:** Rules to verify regulatory links using gene expression data

TF Expression	Gene Expression	Result	
		(+)	(-)

Yes	Yes	*C*	
Yes	No		*C*
No	Yes	*N*	*C*
No	No	--	

The predicted regulatory links, of type positive (+) or negative (-) regulation, are confirmed using the gene expression data based on a set of rules. *C* represents the confirmed links,  represents the contradictory links and *N* represents the neutral links.

For a positive gene regulation type (+) in a regulatory link, the TG should be expressed if the TF is expressed in an experiment. Therefore, if both the TF and TG from a predicted regulatory link are expressed in an experiment then this experiment is classified as Confirming. If the TF is expressed but the TG is not, then the experiment is considered to be Contradictory because the expressed TF is not able to express the TG as it should in the case of positive gene regulation. For a regulatory link, if the TG is expressed but the TF is not, then the experiment is marked as Neutral because it does not confirm or contradict the regulatory link. This experiment does allow that the TG may be regulated by other TFs as well. The TF not expressed in the experiment might be expressed in another experiment supporting the regulatory link. Additionally, if either the TF or the TG from a predicted regulatory link is not expressed in an experiment, then nothing can be inferred and that experiment is ignored for that regulatory link.

In the case of negative gene regulation (-), the TG should not be expressed if the TF is expressed in the experiment. Accordingly, if both the TF and the TG from a predicted regulatory link are expressed or are not expressed, then the experiment is marked Contradictory. But for a predicted regulatory link, if the TF is expressed and the TG is not, or if the TF is not expressed and the TG is expressed, then the experiment is considered to be Confirming that regulatory link.

Finally, each regulatory link is analyzed by identifying the number of times that link has been Confirmed, Contradicted and Neutral in all the gene expression experiments used. These values are then compared in different ways to evaluate the results for the regulatory links. This counting-based approach is needed because the limited number of experiments normally available for non-model organisms are usually insufficient to predict regulatory links from the expression data.

### Limitations of regulatory network mapping

Using the method for regulatory network mapping described in the previous subsection, only transcription gene regulatory network data is being mapped from one genome to another, and this does not include any information about the post-transcription regulation process.

It is important to understand how many regulatory links are conserved over different genomes since there is no distinct definition of these. Many regulatory links from the source genome cannot be mapped to the target genome for various reasons. Firstly, both the TF and the TG in a regulatory link might not have similar TFs and TGs at all in the source genome, depending on how different the genomes are. Secondly, many similar TFs or TGs present in the source genome might not have their corresponding similar TFs or TGs present in the target genome. Thirdly, TFBS motifs need not be conserved well among genomes due to changes in the sequences over time. Fourthly, not all source genome TGs contain the TFBS motifs of their regulator (TF) in the available regulatory network used for mapping.

Additionally, some binding sites are present in the nucleotide sequences but are not involved in the regulation of genes in certain conditions by being inactive [23]. These binding sites can be inactive due to the occupancy of nucleosomes hindering the binding of the TF to the binding site in any genome. About 75 to 90 percent of the DNA in a genome is bundled up in nucleosomes. The unwrapped DNA stretching between two neighboring nucleosomes is called linker DNA. Hence, binding sites present on the linker DNA are largely the active binding sites available to interact with the proteins to form protein complexes.

It has been estimated in a research study [24] that there might be 0 to 200 possible TGs for a TF in *S. cerevisiae*, though approximately only 3 percent of the TGs will have their binding sites bind to these TFs of *S. cerevisiae*. Similar density of binding between TGs and TFs is also found in higher eukaryotes. This suggests that gene expression data can only support the three percent of the possible regulatory relationships that actually exist at a certain time. Some binding sites may not even be involved in the gene regulation process and have no change in their gene expression values in two different treatments. These binding sites might be conditional relying on the presence or absence of other TFs.

It is also shown [24] that binding patterns for some TFs are dynamic and change under different environmental conditions. Also, all the predicted regulatory links may not necessarily be the direct links in the target genome [24], and so the TGs can be regulated indirectly by the TFs based on the feedforward loop motif concept. In feedforward motifs, one TF regulates another TF, and they both regulate a TG, though the regulation between the first TF and the TG is at least partly an indirect regulation. It is extremely difficult to predict indirect regulatory links without using substantial gene expression data, and so far only limited information about the direct regulatory links is available for the model organisms.

All the above facts show that gene regulatory networks are very complex networks and it is extremely difficult to integrate all the different factors mentioned earlier to determine regulatory networks, even for a model organism.

## Results

### Transcription factor mapping

The TF mapping result sets from *S. cerevisiae* to *A. thaliana* are shown in Table 2. There are 35351 nucleotide sequences [25] used for TF mapping, and 1922 TFs [26] for *A. thaliana* used to analyze the results.

Even though *S. cerevisiae* is a much smaller genome than *A. thaliana*, many confirmed TFs have been identified for *A. thaliana*. A large number of nucleotide sequences have been determined by this method to not be TFs, thus contributing to the high value of true negatives. The number of mapped links decreases more from set TFf to set TFsf than from set TFbl to set TFf. The number of distinct TFs mapped decreases from set TFbl to set TFf by 333 and from set TFf to set TFsf by 316. This indicates that the results are refined from set TFbl to TFf when protein family classification is used along with sequence similarity for TF mapping. Protein subfamily classification further refines the results from set TFf to TFsf. Comparing results in set TFbl and set TFf, there is a large decrease of 261 in false positives and a low decrease of 72 in true positives from set TFbl to set TFf. Therefore, set TFf is better than set TFbl by refining the BLAST results based on family classification. There is also a low decline in the percentage of available TFs correctly identified in set TFbl and set TFf. This indicates that mapping TFs with sequence similarity and protein family classification gives better results than using only sequence similarity. Comparing results in set TFf and set TFsf, there is a huge decline of 237 in true positives and a low decrease of 79 in false positives from set TFf to set TFsf. This indicates that large number of true TFs are lost with only a few wrongly mapped TFs discarded from set TFf to set TFsf. Therefore, with a huge decline in true positives and in the percentage of available TFs correctly predicted, and a low decrease in false positives, set TFf has better results than set TFsf. This suggests that the refined results in set TFsf that are obtained using protein subfamily classification are not better than the results in set TFf based on protein family classification. True negatives in all the mapped TF sets are very high because most of the sequences that are not TFs have very low similarity to the TF sequences.

**Table 2:** TP, FP, FN and TN values for the mapped TFs in the target genome

	TFbl	TFf	TFsf
**Number of mapped links**	1344	903	212
**Number of mapped TFs**	767	434	118
**True Positives (TP)**	400	328	91
**False Positives (FP)**	367	106	27
**True Negatives (TN)**	33062	33323	33402
**False Negatives (FN)**	1522	1594	1831

Comparison of the three result sets of predicted transcription factors (TFs) identified for the *A. thaliana* genome based on binary classifier. TFf result set is analysed as the preferred set for transcription factor mapping. E-value threshold used for BLAST is 1e-1.

### Target gene mapping

The five sets of results for TGs mapped from *S. cerevisiae* to *A. thaliana* are shown in Table 3. There are 25191 TGs present in the available binding site data of *A. thaliana*[*27*] used for analyzing the results.

**Table 3:** TP, FP, FN and TN values for the mapped TGs in the target genome

	TGbl	TGbs	TGblbs	TGpr	TGgalf
**Number of mapped TGs**	2252	35181	2252	25259	25388
**True Positives (TP)**	1717	25180	1717	24973	25077
**False Positives (FP)**	535	10001	535	286	311
**True Negatives (TN)**	9625	159	9625	49	24
**False Negatives (F)**	23474	11	23474	218	114

Comparison of the five result sets of predicted target genes (TGs) determined for the *A. thaliana* genome based on binary classifier. TGpr and TGgalf are analyzed as the preferred sets for target gene mapping. E-value threshold used for BLAST is 1e-1

The first three sets, TGbl, TGbs and TGblbs, use the nucleotide sequence data of *A. thaliana* as the target genome database for searching the target genes. The first set TGbl is obtained as a result of using BLAST only, the first step in the TG mapping method. The second step in the TG mapping method, which includes searching TFBS motifs, is used to identify the second set TGbs. To refine these results, BLAST is used first to find highly similar TGs before searching for the TFBS motifs. To refine these results, BLAST is used first to find highly similar TGs before searching for the TFBS motifs. Hence, the third set TGblbs is determined using both BLAST and searching TFBS motifs. Results for set TGbs show that most of the *A. thaliana* sequences are predicted as TGs containing TFBS motifs in the target genome. True positives are the highest for this set, among the first three sets, along with low false negatives. However, it does have quite a high false positive count.

Comparing set TGbl and set TGblbs, the same set of target genes are identified in both result sets. These results show that the TFBS motifs were found in all the highly similar TGs identified using BLAST. Hence, no true TG is lost when the predicted TGs (set TGbl) determined using BLAST are refined further by searching the TFBS motifs in the nucleotide sequences, resulting in set TGblbs.

The last two sets, TGpr and TGgalf, use the promoter sequence data of *A. thaliana* as the target genome database for searching the target genes. The fourth set TGpr is determined by searching TFBS motifs in the target genome database. The last set TGgalf is obtained by using the GALF-P tool (Genetic Algorithm with Local Filtering and adaptive post-processing techniques) to identify TFBS motifs, followed by searching these TFBS motifs in the target genome database. A large number of target genes are identified in set TGpr and set TGgalf with lower false positives as compared to set TGbs. This decrease in false positives reflects the increased selectivity of the promoter sequence data used for sets TGpr and TGgalf instead of the nucleotide sequence data used for set TGbs. This improvement in accuracy over TGbs, TGbl and TGblbs makes TGpr and TGgalf the best sets for mapping target genes. There is little difference between the results for these two sets.

Almost all the TGs are identified in *A. thaliana*, but to work properly for mapping regulatory links these predicted TGs need to be the correctly mapped TGs linking to the right TF. This will be verified in the analysis of predicted regulatory links discussed in the following subsection.

### Mapped regulatory elements integration and confirmation

The two sets of TFs based on the same family (TFf) and subfamily (TFsf), identified in the first step of regulatory network mapping, are integrated with the four sets of predicted TGs (TGbs, TGblbs, TGpr, TGgalf) determined in the second step. The results of regulatory elements integration consist of six sets of regulatory links mapped from *S. cerevisiae* to *A. thaliana* as shown in Table 4. There are 14254 regulatory links present in the available regulatory network [28] of *S. cerevisiae*. The predicted regulatory links for *A. thaliana* are then analyzed using gene expression data [24] to classify the gene expression experiments for each regulatory link into Confirming (*C*), Contradictory (), and Neutral (*N*) groups based on the rules described in the previous section. The total number of Confirming, Contradictory and Neutral values for a regulatory link to be analyzed should be equal to the number of gene expression experiments of *A. thaliana* used. Then the Confirming (*c*), Contradictory (), and Neutral (*n*) values are compared in different ways in Table 4 to analyze the predicted regulatory links using the 43 gene expression experiments for *A. thaliana*.

**Table 4:** Regulatory links confirmed for *A. thaliana* using gene expression data

	TFsf-TGblbs	TFf-TGblbs	TFsf-TGbs	TFsf-TGpr	TFf-TGpr	TFf-TGgalf
**Number of mapped TFs**	118	434	118	118	434	434
**Number of mapped TGs**	2252	2252	35181	25259	25259	25388
**Number of links mapped**	43423	480524	536154	274400	3182551	242030
**Number of links analyzed**	3056	8621	6085	34529	236585	25227
	2090	2628	2995	2343	93287	3727
	2109	2375	2968	2323	101224	2012
	58	344	30	26	12737	1715
	103	215	100	66	19037	503
	786	5687	2987	2050	87983	150

Comparison of the six result sets of predicted regulatory links mapped from the *S. cerevisiae* genome to the *A. thaliana* genome. For every result set, the number of confirmed regulatory links is compared with the number of contradicted and neutral regulatory links to analyze the results. TFsf-TGblbs is the preferred set for regulatory links mapping.

In the fifth row, weights of two and one are assigned to the Contradictory and Neutral values to obtain a threshold for comparing it to the Confirming value. If the Confirming value is higher than this threshold for a regulatory link, then the link is considered to be a verified true regulatory link mapped in the target genome. This is done to ignore any error due to outliers in the gene expression data, and to require that a high proportion of the results for a link be Confirming, since the Confirming experiments need to outweigh not just the Contradictory experiments but also the Neutral ones. It is also evaluated based on the ratio between the Confirming and Contradictory values for the regulatory links, to determine for which links there is much greater evidence to support it than there is to contradict it. The Confirming value is compared with thrice and twice the value of Contradictory in the sixth, seventh and eighth rows of Table 4. The regulatory links satisfying these conditions are well supported by the gene expression experiments. In the ninth row, the number of regulatory links with a Confirming value higher than or equal to their Contradictory value but lower than twice the Contradictory value are determined. The regulatory links that meet this condition are not as well supported by the expression data. The Ninth row shows the number of regulatory links that have a Contradictory value higher than their Confirming value. These regulatory links are not supported by the gene expression data.

Comparing set TFsf-TGblbs and set TFf-TGblbs, two-third of the analyzed regulatory links are verified as true regulatory links for set TFsf-TGblbs, whereas only one-third of the analyzed regulatory links are verified for set TFf-TGblbs. Therefore, the results from the TFsf-TGblbs set are better, with a much higher percentage of correctly mapped regulatory links in the target genome.

Comparing set TFsf-TGblbs and set TFsf-TGbs, the percentage of regulatory links analyzed with a Contradictory value more than the their Confirming value increases from approximately 25 percent to about 50 percent from set TFsf-TGblbs to set TFsf-TGbs. This indicates that half of the regulatory links analyzed in set TFsf-TGbs are definitely not supported by the gene expression experiments.

Less than one-fifth of the regulatory links have been verified as true regulatory links in the set TFsf-TGpr and set TFf-TGgalf. Comparing set TFsf-TGblbs and set TFf-TGpr, the correctly mapped regulatory links are about two-third of the analysed links in the set TFsf-TGblbs and about one-third in the set TFf-TGpr. The percentage of regulatory links analyzed with a Contradictory value more than the their Confirming value increases from approximately 25 percent to about 39 percent from set TFsf-TGblbs and set TFf-TGpr.

Hence, set TFsf-TGblbs has better results than the other regulatory link sets, based on having a higher percentage of true regulatory links identified and a lower percentage of links contradicted more than they are confirmed. This set also shows that, even though *S. cerevisiae* and *A. thaliana* are two organisms that are evolutionarily far apart, they still do share a significant amount of regulatory information among them.

## Discussion

The TF mapping result set TFf comprises the best results when we only consider how many TFs are mapped, but it does not produce the best regulatory links set when integrated with the mapped TG set TGblbs. This indicates that the set TFsf containing the mapped TFs based on sequence similarity and subfamily classification contains the most efficiently and correctly mapped TFs for the purpose of mapping regulatory links.

The TGbs set, TGpr set and TGgalf set contain the best results of mapped TGs from the previous subsection, but they do not work well when used in the integration of regulatory elements to predict regulatory links. The additional predicted TGs in these sets lead to too many false regulatory links in the sets TFsf-TGbs, TFf-TGpr, TFsf-TGpr, and TFf-TGgalf. These false links indicate that many of the true TGs identified for the target genome in set TGbs, set TGpr and set TGgalf are, however, not the correctly mapped TGs linked to the right TF in their corresponding regulatory link result sets. All the predicted TGs do contain the TFBS motifs for some TF but these TGs need to correspond to the correct TF, identifying the right regulatory link in the target genome. These results suggest that, in order to be used to map regulatory links, the TGs identified in the target genome using TFBS motifs also need to be similar in sequence to the source genome TGs, as identified in set TGblbs. Therefore, the result of other possible sets, TFf-TFbs and TFsf-TGgalf, are not included in this paper as their sets of regulatory elements are already determined to lead to many false positives when integrated to produce a predicted regulatory link.

## Conclusions

A three step approach has been proposed to map a regulatory network from a model organism to a non-model organism. This includes mapping the transcription factors and the target genes separately and then integrating these regulatory elements to identify the regulatory relationships for the target genome. Rules are established to evaluate the predicted regulatory links using gene expression data from the target genome.

Results are obtained in the transcription factor mapping step based on three techniques, using BLAST, using BLAST with protein family classification and using BLAST with protein subfamily classification. Results show that the technique based on sequence similarity and protein family classification maps transcription factors most efficiently from *S. cerevisiae* to *A. thaliana*; therefore, it is the preferred method for general transcription factor mapping.

There are five techniques used for target gene mapping, based on sequence similarity, TFBS motifs, and sequence similarity along with TFBS motifs. Using three methods, most of the target genes are identified correctly for *A. thaliana* by searching TFBS motifs only. These methods have better results with many more true positives than using only sequence similarity and than using sequence similarity with TFBS motifs. The same set of target genes is predicted using sequence similarity and using sequence similarity along with TFBS motifs. Therefore, the methods using TFBS motifs only are the preferred method for general target gene mapping. Among these three methods, the two methods using the target genome promoter sequence database for searching TFBS motifs are better with much lower false positives.

Six sets of regulatory links are obtained in the regulatory elements integration step. The first set combines the mapped transcription factors based on sequence similarity and protein family classification with the target genes based on sequence similarity and seaching TFBS motifs in the nucleotide sequence database. The second set combines the mapped transcription factors based on sequence similarity and protein subfamily classification with the target genes based on sequence similarity and seaching TFBS motifs in the nucleotide sequence database. The mapped transcription factors based on sequence similarity and protein family classification are integrated with the target genes based on seaching TFBS motifs in the nucleotide sequence database in the third set. The fourth set combines the mapped transcription factors based on sequence similarity and protein subfamily classification with the target genes based on searching TFBS in the promoter sequence database. The mapped transcription factors based on sequence similarity and protein family classification are integrated with the target genes based on seaching TFBS motifs in the promoter sequence database in the fifth set. The sixth set combines the mapped transcription factors based on sequence similarity and protein family classification with the target genes based on identifying and then searching TFBS motifs in the promoter sequence database. In the results, the large amount of target genes identified using the preferred method for target gene mapping produce many false regulatory links, since, while they are target genes, they are not linked to the correct transcription factor.

Additionally, the transcription factors from the preferred method of transcription factor mapping also contribute to many false regulatory links when used in the regulatory elements integration step.

Hence, the predicted regulatory links obtained by integrating the mapped transcription factors based on sequence similarity and protein sub-family classification and mapped target genes based on sequence similarity and TFBS motifs contain the most regulatory links for the target genome that are verified by the gene expression data. This implies that more correctly mapped target genes that link to the right transcription factor are determined by using BLAST along with the TFBS motifs. Also, the correctly mapped transcription factors are obtained using the method based on sequence similarity and protein sub-family classification. This suggests that regulatory relationships are conserved between different genomes and can be mapped between them. Therefore, for a newly sequenced organism, a related model organism can be used to determine some regulatory information for the lesser explored organism, avoiding the need to complete significant expression experiments for the target organism.

The use of TFBS information in finding target genes, while showing the best final results, does cause some errors. Even when the target genes contain the regulatory motifs of certain transcription factors, these genes may not be regulated by these transcription factors at all. This is because, only a very small percentage of binding sites are available to bind to interact with the transcription factors, as mentioned earlier in the limitation of regulatory networks section. False positives in our results could then be reduced by integrating information about the active binding sites in the target genes. Considering alternative sources of TFBS information could also decrease the false negatives, as we discovered that the lack of an instance of the appropriate binding site motif did not always correlate to lack of regulation in the source genome.

More biological and regulatory information can also be integrated further into the regulatory network mapping method as more data becomes available for non-model organisms. Experimental data, such as gene expression, time series, or flow cytometry data, can be used to filter predicted links, even if this data includes only a small number of experiments. Furthermore, additional information about gene regulation at different stages of gene expression can be incorporated as well.

## Competing interests

The authors declare that they have no competing interests.

## Authors' contributions

RS designed and carried out the experiments, and drafted the paper. PAE was responsible for directing the experiments and contributed to the discussion of the method and results. Both PAE and VCB supervised the work and assisted in polishing the final paper.
